# Neurodegenerative diseases: from gut-brain axis to brain microbiome

**DOI:** 10.3389/fnagi.2023.1171955

**Published:** 2023-05-19

**Authors:** Robert P. Friedland, Bodduluri Haribabu

**Affiliations:** ^1^Department of Neurology, University of Louisville School of Medicine, Louisville, KY, United States; ^2^Department of Microbiology and Immunology, University of Louisville School of Medicine, Louisville, KY, United States

**Keywords:** dementia, amyloid, Alzheimer's, microbiota, Aducanumab

## Introduction

The proportions of global populations that are over 65 years of age are growing rapidly. In Japan there are now 2.5 times more diapers sold for adults than for children. Because the neurodegenerative disorders Alzheimer's, Parkinson's and amyotrophic lateral sclerosis (ALS) are all linked to aging the number of cases with these conditions are expanding greatly. It is estimated that there are 55 million people currently with dementia in the world and it is projected that there will be 139 million globally with dementia by 2050. Despite intense efforts by scientists and drug companies worldwide there is still no disease modifying therapy for Alzheimer's disease (AD), the most common cause of dementia.

On June 16, 2022, it was announced that an anti-amyloid antibody, Crenezumab, failed in a large clinical trial. This result followed the complex story of another anti-amyloid antibody, Aducanumab. In June 2021, the US Food and Drug Administration (FDA) granted accelerated approval to Aducanumab, developed by Biogen, for the treatment of AD. Randomized placebo-controlled studies had shown that the antibody removed amyloid deposits in the brain. These plaques of aggregated molecules have been an important finding in AD for over 120 years. Despite this encouraging development, the drug did not provide clinically meaningful cognitive benefit. And disturbingly, over 40% of subjects experienced potentially hazardous side effects involving brain swelling. Because of these findings an FDA scientific advisory panel had recommended in November 2021, by a vote of 10 to 0 (with one voting “uncertain”), that the antibody should not be approved.

Responses to the FDA's approval of the Aducanumab have been overwhelmingly negative. Three members of the FDA's advisory panel which recommended against approval resigned following the FDA's decision to approve the antibody. Biogen was criticized for the cost of $56,000 for 1 year of treatment, and they have lowered the cost to $28,000. In response to the high cost and lack of efficacy the European Medicines Agency has refused marketing authorization for Aducanumab and the US Centers for Medicare and Medicaid Services will pay for the antibody only for patients enrolled in qualifying clinical trials (Center for Medicare and Medicaid Services, 2022). In addition, the US Veterans Administration will not include the drug in its formulary because of its lack of effectiveness.

There have now been over 20 studies that show that reducing amyloid in the brain is not helpful in significantly improving the clinical outcome in persons with AD (see Imbimbo et al., 2020, for review). The failure of Crenezumab and Aducanumab to provide clinical benefit is the latest of a series of failures in AD treatment trials going back several decades. Lecanemab, a humanized immunoglobulin gamma 1 (IgG1) monoclonal antibody directed against aggregated soluble and insoluble forms of amyloid beta, has also been shown to have limited cognitive benefit. Of concern also is the risk of brain edema, hemorrhage and death associated with these agents (Table 1). Their use will require careful monitoring, genetic testing and exclusion of patients suspected of having cerebral amyloid angiopathy or taking anticoagulants.

**Table 1:** Side effects of anti-amyloid monoclonal antibody therapy.

• ARIA-E cerebral edema • ARIA-H small and large brain hemorrhages • Superficial siderosis • Cortical atrophy

ARIA, Alzheimer related imaging abnormality.

We are concerned that these disappointing therapeutic developments have obscured recent advances in our understanding of the role of the microbiota in the molecular mechanisms of Alzheimer's disease.

## The microbiota and neurodegeneration

Research in the last 10 years has shown that we are all home to a complex community of microorganisms, referred to as the microbiota. Considerable evidence demonstrates that they are highly involved in the initiation and maintenance of disease mechanisms in AD and related disorders.

The microbiota are composed of bacteria, fungi, viruses and other microbes which live on our body surfaces, as well as inside us (Parker et al., this issue, hyperlink). The number of our own cells is similar to the number of these partner organisms and they contain over a hundred times more genetic information than does our own DNA. They are with us at every moment of our lives, beginning shortly after birth. All of our ancestors going back 1 billion years also had microbial companions. *This means that we evolved with them and they evolved with us*.

This co-evolution is key to understanding their importance in our lives. The microbiota evolved the capacity to enhance the tolerance of our immune system to their presence. This acceptance allows them to thrive without causing an aggressive immune response from the host which would be damaging. At the same time, our immune system evolved the capacity to monitor the presence of our microbial communities and control our immune responses, so that a symbiotic relationship, which benefits us both, can be maintained. It has been shown that the microbiota are key to the healthy development of the immune system. They also play important roles in metabolism nutrition learning memory and protection from disease causing organisms (Bostick et al., 2022).

A critical role of the microbiota in AD and the related neurodegenerations such as Parkinson's diseases (PD), and ALS has been recently demonstrated (Klann et al., Li et al., Shen et al., this issue, hyperlinks, Kurlawala et al., 2023). Key features of all these conditions include deposits of aggregated proteins (such as the amyloid β protein in AD and alpha synuclein in PD), activation of the immune system in the brain and the presence of reactive oxygen molecules, which are unstable, highly reactive and destructive. The microbiota in the gut influence all of these processes through diverse molecular mechanisms, some of which are being determined in studies conducted in labs around the world.

## Microbial amyloid proteins

Microbes in the gut, including the nose, mouth, and intestines make functional bacterial amyloid proteins which have been shown to cause templated cross-seeding of neuronal proteins in the brain, accelerating cerebral amyloid deposition in neurodegenerative disease models (Sampson et al., 2020). Otzen et al. in Denmark have shown that the microbiota make functional amyloid proteins that may be involved in neurodegeneration (Christensen et al., 2021). Landau et al. in Haifa have demonstrated the cross-seeding of the amyloid beta protein by curli *in vitro* (Perov et al., 2019). Importantly, passage of amyloid proteins from the gut to the brain via the vagus nerve has been documented in rats by Holmqvist et al. (2014), as discussed in this special issue by Geng et al. (hyperlink). A genome wide screen of Wang et al. (2021) from Hong Kong showed that the functional bacterial amyloid protein curli made by *Escherichia coli* and other bacteria colocalized with neuronal amyloid inside neurons, promoted aggregation though cross-seeding and promoted disease in worm models of AD, ALS and Huntington's.

Furthermore, functional bacterial amyloids proteins in the gut are recognized as pathogen associated molecular patterns by the innate immune system, which has been shown to increase the immune response to neuronal amyloids in the brain and worsen functional deficit (Chen et al., 2016; Friedland and Chapman, 2017). It has been shown that functional bacterial amyloids activate Toll-Like receptors 1 and 2, NFkB and iNOS leading to enhanced inflammation and oxidative toxicity. This is a similar pathway by which neuronal amyloids are recognized by the brains immune system (Friedland and Chapman, 2017). Heneka and associates in Bonn, Germany, have shown that activation of the innate immune system in the brain contributes to aggregation of the amyloid beta protein in AD model mice (Ravichandran and Heneka, 2021).

## Therapeutic potential of microbiota in AD

An exciting feature of this work is that our microbial partners are relatively easy to adjust. They are our captives and must eat what we give them. Studies show that a change in diet in humans in as little as 2 weeks can significantly change the nature of bacterial populations in the intestine with beneficial effects on health (O'Keefe et al., 2015). This effort to improve our health through changes in diet can be referred to as “gene therapy in the kitchen,” because a change in diet changes our internal bacterial communities and their DNA. it is critical that we be aware of the influence of nutrition on our microbiota (Friedland, 2022).

## Discussion

Furthermore, considerable effort is being extended globally to develop agents which will affect the gut microbiota and their metabolic products with benefits for our health and fitness (Table 2). It is clear that there will be drugs which influence gut bacteria to provide benefit for the brain, without the need for the drug itself to enter the blood or cross the blood-brain barrier. There are many potential avenues for exploration of this therapeutic approach: probiotics provide live bacteria; prebiotics provide food for desirable bacteria, and postbiotics are beneficial products produced by the microbes (Kim et al., this issue, hyperlink). Antibiotics also have strong influences on gut bacteria, as shown by Liu et al. in this issue (hyperlink). Bacterial transplants are being explored and agents which alter bacterial metabolism with benefits for health are being developed (Zhang et al., 2021; Wang et al. both from this issue, hyperlinks). For example, Sampson et al. (2020) has shown that oral intake of polyphenols with anti-amyloid effects have beneficial influences in Parkinson model mice. In this issue Chung et al. (hyperlink) reports the influence of the polyphenol resveratrol on the gut-brain axis.

**Table 2:** Potential microbial based therapies.

• Prebiotics, probiotics, synbiotics, postbiotics • Curds • Fecal microbiota transplants (FMT) • Phages • Diet • Dental care • Antibiotics • Antibodies • Vaccines • Medical foods • Amyloid inhibitors (e.g., polyphenols)

Ninety to ninety-nine percent of cases of neurodegenerative diseases are not caused by genes. The microbiota may be a major environmental factor influencing the course of their development. The amyloid deposits in the brain, which anti-amyloid treatments remove, are a biomarker of AD, not the disease itself. The papers in a recent special issue of *Frontier of Aging Neuroscience* provide good reasons to believe that new approaches based on microbial mechanisms will powerfully influence the critical primary pathways of disease development with powerful effect (Tetz, 2022).

## Author contributions

All authors listed have made a substantial, direct, and intellectual contribution to the work and approved it for publication.
